# Comparison of Rehabilitation Outcomes for Long Term Neurological Conditions: A Cohort Analysis of the Australian Rehabilitation Outcomes Centre Dataset for Adults of Working Age

**DOI:** 10.1371/journal.pone.0132275

**Published:** 2015-07-13

**Authors:** Lynne Turner-Stokes, Roxana Vanderstay, Tara Stevermuer, Frances Simmonds, Fary Khan, Kathy Eagar

**Affiliations:** 1 King’s College London, School of Medicine, Department of Palliative Care, Policy and Rehabilitation, London, United Kingdom; 2 University of Wollongong, Australasian Rehabilitation Outcomes Centre / Australian Health Services Research Institute, Wollongong, Australia; 3 University of Melbourne and Royal Melbourne Hospital, Department of Rehabilitation, Melbourne, Australia; Nathan Kline Institute and New York University School of Medicine, UNITED STATES

## Abstract

**Objective:**

To describe and compare outcomes from in-patient rehabilitation (IPR) in working-aged adults across different groups of long-term neurological conditions, as defined by the UK National Service Framework.

**Design:**

Analysis of a large Australian prospectively collected dataset for completed IPR episodes (n = 28,596) from 2003-2012.

**Methods:**

De-identified data for adults (16–65 years) with specified neurological impairment codes were extracted, cleaned and divided into ‘Sudden-onset’ conditions: (Stroke (n = 12527), brain injury (n = 7565), spinal cord injury (SCI) (n = 3753), Guillain-Barré syndrome (GBS) (n = 805)) and ‘Progressive/stable’ conditions (Progressive (n = 3750) and Cerebral palsy (n = 196)). Key outcomes included Functional Independence Measure (FIM) scores, length of stay (LOS), and discharge destination.

**Results:**

Mean LOS ranged from 21–57 days with significant group differences in gender, source of admission and discharge destination. All six groups showed significant change (p<0.001) between admission and discharge that was likely to be clinically important across a range of items. Significant between-group differences were observed for FIM Motor and Cognitive change scores (Kruskal-Wallis p<0.001), and item-by-item analysis confirmed distinct patterns for each of the six groups. SCI and GBS patients were generally at the ceiling of the cognitive subscale. The ‘Progressive/stable’ conditions made smaller improvements in FIM score than the ‘Sudden-onset conditions’, but also had shorter LOS.

**Conclusion:**

All groups made gains in independence during admission, although pattern of change varied between conditions, and ceiling effects were observed in the FIM-cognitive subscale. Relative cost-efficiency between groups can only be indirectly inferred. Limitations of the current dataset are discussed, together with opportunities for expansion and further development.

## Introduction

In 2005, the UK Department of Health published a National Service Framework (NSF) for long term neurological conditions (LTNC)[1]. Previous NSFs had already focused specifically on Older Adults and on Children, so the primary focus of the NSF for LTNC was on adults of working age (predominantly 16–65 years). Because of the diversity of presentation and the many diagnoses covered by this term, the NSF took a novel approach to the classification of neurological conditions, grouping them by pattern of presentation as follows:

- Sudden-onset conditions e.g. stroke, brain and spinal cord injury, acute polyneuropathies (e.g. Guillain-Barré syndrome (GBS)).
- Progressive conditions e.g. multiple sclerosis (MS), Parkinson’s Disease (PD), motor neurone disease (MND), chronic demyelinating polyneuropathies.
- Stable conditions (with changing needs due to development or ageing), e.g. cerebral palsy (CP), post-polio.

Although rehabilitation outcomes are well described in the literature for the Sudden-onset category–particularly for stroke and traumatic brain injury[2, 3], they are less well described for other neurological conditions (such as PD, neuropathies or cerebral palsy). One reason for this is the small number of patients within each diagnostic group. By combining patients with similar presentations into one larger group, it may be possible to explore outcomes and draw conclusions on a broader evidence base than can be achieved for each condition individually. Exactly how conditions should be grouped within those broad categories, however, remains open to question.

The analysis of prospectively-collected datasets provides an important opportunity to evaluate and compare outcomes across different conditions. Although cohort analyses do not provide direct evidence of the effectiveness of rehabilitation, they can afford more detailed information about which types of patients benefit from which types of treatment and in what ways[4, 5]. Importantly, they furnish generalisable information about the changes that occur in the course of real-life clinical practice (practice-based evidence), which is of interest to providers and purchasers of rehabilitation services[6]. On the other hand, the findings must be interpreted with a degree of caution where there are less rigorous standards for data collection, or in health settings where reimbursement is dependent on the demonstration of functional gain.

In Australia, there is no direct link between outcome and payment for rehabilitation services. The Australasian Rehabilitation Outcomes Centre (AROC) holds a large centralised database, which gathers a standard set of information on both process and outcomes for every person admitted for inpatient rehabilitation[7]. Established in 2002 as a joint initiative of the Australian rehabilitation sector (providers, payers, regulators and consumers), the dataset comprises case episode data for admissions for rehabilitation from participating services across Australia and New Zealand (currently almost 950,000 episodes of care from 266 facilities). The database provides a national benchmarking service as well as providing information to improve understanding of factors that influence quality of care and patient’s rehabilitation outcomes.

In the UK, an equivalent national dataset for specialist neurorehabilitation has been developed through the UK Rehabilitation Outcomes Collaborative (UKROC). The UKROC dataset represents the In-patient Rehabilitation module of the Long Term Neurological Conditions Dataset[8]. The design is modelled closely on the AROC dataset, but extends it in some areas. As the database is still in development, there is opportunity to learn from analyses of other large datasets to determine what further information may need to be collected alongside the core data, in order to address the critical questions in neurological rehabilitation over the coming decade. Crucial to the success of clinical datasets, however, is the engagement of clinicians to ensure that data are as complete and as accurate as possible. They need a frame of reference against which to compare their experience, and to gauge their outcomes in treating not only for the common conditions, but also for the rarer ones.

The primary objective of this analysis is to describe and compare outcomes for in-patient rehabilitation across a wide range of long-term neurological conditions in working-aged adults, which is the predominant focus for many of the more specialised rehabilitation services (Level 1 and 2) contributing to the UKROC database[9].

- We wished to explore how episode data might reasonably be collated into groups according to the NSF definitions for future analyses, and to present the information in a form that is meaningful to clinicians.
- We also wished to determine the extent to which the AROC dataset could be used to distinguish and describe these different groups.

The results will also assist with the identification of any additional information or approaches that should be included in future versions of the Australasian and UK datasets to align them more closely.

## Materials and Methods

### The AROC dataset

The full AROC dataset includes 42 items: socio-demographic, medical (impairment codes, co-morbidities, complications), episode items (admission dates), funding and employment details, and outcome data (patient level of function at admission and discharge)[10]. Within the AROC dataset, there are four principal ‘Impairment categories’ for neurological conditions. Each category is further subdivided into specified ‘Impairment codes’ (see Table 1).

**Table 1:** Complete and incomplete case episodes categorised by Impairment code.

Impairment category	Code	AROC Impairment Code Descriptor	CompleteEpisodes	IncompleteEpisodes
			Frequency(%)	Frequency(%)
**Stroke**	1.1	Left body involvement (Right brain)	5024 (17.6)	1175 (14.5)
	1.2	Right body involvement (Left brain)	4874 (17.0)	1082 (13.4)
	1.3	Bilateral cerebrovascular accident (CVA)	534 (1.9)	149 (1.8)
	1.4	No paresis	527 (1.8)	114 (1.4)
	1.9	Other CVA	1568 (5.5)	414 (5.1)
**Brain dysfunction**				
Non-traumatic	2.11	Sub-arachnoid haemorrhage (SAH)–non-traumatic	1014 (3.5)	284 (3.5)
	2.12	Anoxic brain damage	321 (1.1)	133 (1.6)
	2.13	Other non-traumatic brain dysfunction	1879 (6.6)	656 (8.1)
Traumatic	2.21	Traumatic–open injury	456 (1.6)	174 (2.2)
	2.22	Traumatic–closed injury	3895 (13.6)	1371 (17.0)
**Neurological conditions**				
	3.1	Multiple sclerosis (MS)	2292 (8.0)	384 (4.8)
	3.2	Parkinsonism (PD)	847 (3.0)	96 (1.2)
	3.3	Polyneuropathy	203 (0.7)	70 (0.9)
	3.4	Guillain-Barré Syndrome (GBS)	805 (2.8)	142 (1.8)
	3.5	Cerebral palsy (CP)	196 (0.7)	52 (0.6)
	3.8	Neuromuscular disorders (including MND)	408 (1.4)	111 (1.4)
**Spinal Cord Injury**				
Non-traumatic	4.11	Paraplegia (Complete and incomplete)	1054 (3.7)	418 (5.2)
	4.12	Quadriplegia (Complete and incomplete)	529 (1.8)	259 (3.2)
	4.13	Other non-traumatic spinal cord dysfunction	493 (1.7)	126 (1.6)
			
Traumatic	4.21	Paraplegia (Complete and incomplete)	762 (2.7)	388 (4.8)
	4.22	Quadriplegia (Complete and incomplete)	749 (2.6)	432 (5.3)
	4.23	Other traumatic spinal cord dysfunction	166 (0.6)	46 (0.6)
**Total**			28596	8076

The AROC Impairment codes are not strictly ‘impairments’ according to the World Health Organisation International Classification of Functional Disability and Health (ICF) [12] but a mixture of diagnoses and impairment categories, modelled on the US Uniform Data Systems Impairment codes[3]. In its current form the AROC dataset does not fully support analysis under the different ICF categories.

The primary outcome measure is the Functional Independence Measure (FIM)[11]. The FIM is an 18-item global measure of independence in activities of daily living, subdivided into two subscales: FIM-Motor (13 items covering self-care, sphincters, transfers and locomotion) and FIM-Cognitive (5 items covering communication and social cognition). Each item is scored on a range of 1 (total dependence) to 7 (full independence). AROC holds a territory licence for use of the FIM (a trademark of the Uniform Data System for Medical Rehabilitation, a division of UB Foundation Activities, Inc.) in Australia and New Zealand and is the national certification and training centre for this tool for all accredited rehabilitation clinicians. The following procedures are in place to maximise data quality:

- Clinical staff are required to complete FIM training and to sit a credentialing exam every 2 years.
- All data received by AROC are screened for errors and missing data and, if necessary, the submitting facility is requested to review and correct any inconsistencies.

### Data extraction

For this analysis, we used data gathered from Australian facilities (n = 142) for episodes discharged in the years 2003 to 2012. De-identified data for inpatient rehabilitation (IPR) episodes for adults aged 16–65 years with a specified AROC Impairment Code within the four neurological impairment categories were extracted from the AROC data collection and transferred to SPSS version 18.0 for Windows for analysis. A total of n = 36,672 case episodes were recorded within the period.

For the purposes of our analysis, we were interested in comparing outcomes across the different conditions for those who completed a rehabilitation programme. The first step was therefore to classify episodes into ‘complete’ or ‘incomplete’. Case episodes were considered complete if they met all three of the following criteria:

- Discharged to usual or interim accommodation, or on other non-acute/sub-acute settings (i.e. incomplete episodes were patients who died (n = 68), self-discharged (n = 361), or were transferred back to the acute hospital (n = 4702) or missing data (n = 715)).
- Length of stay (LOS) was between 5–500 days (i.e. incomplete episodes were short admissions for assessment only (n = 1150), very long-stay episodes (n = 13) and missing LOS (n = 54)).
- A valid FIM score (i.e. case episodes with missing or invalid FIM scores (n = 1013) were incomplete).

**Fig 1:**
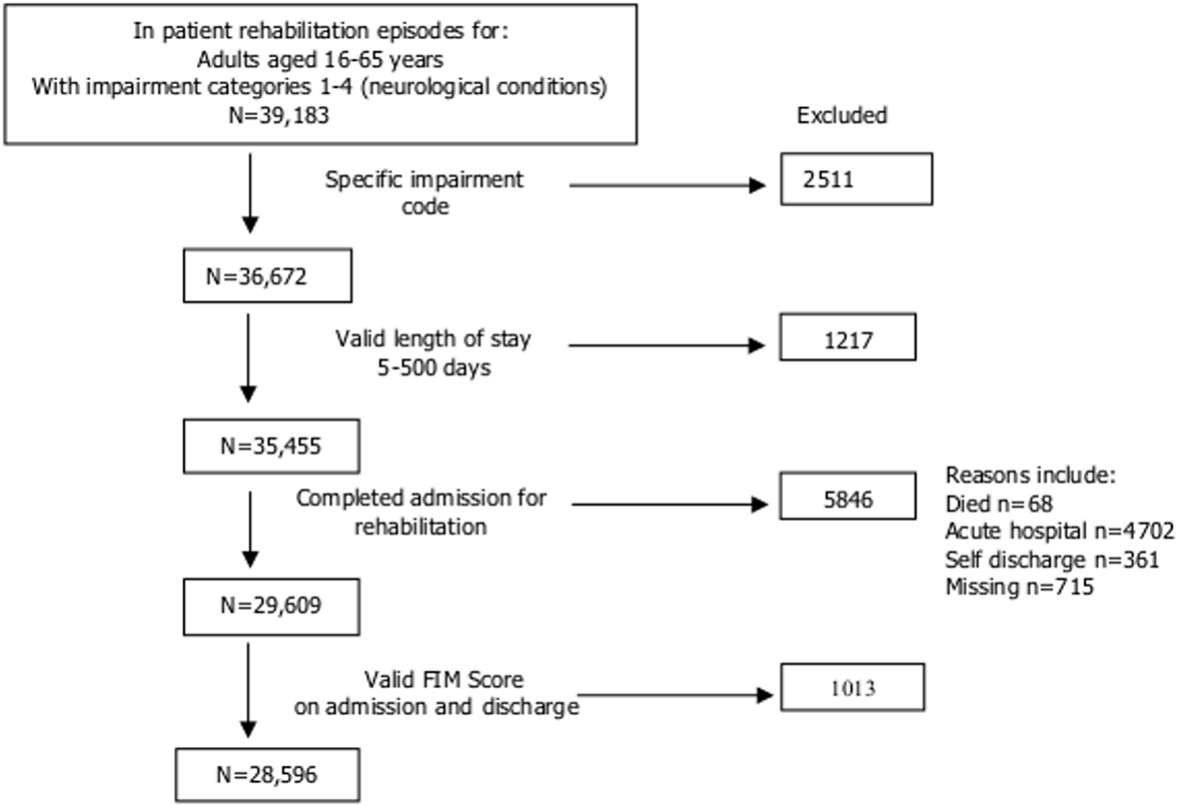
Flow chart—data exclusion. The figure shows the different stages of the data cleaning process to obtain a dataset comprising completed episodes of rehabilitation with valid FIM scores. The most common reason for exclusion, accounting for more than half the excluded cases, was transfer back to the acute hospital setting for medical or surgical management. Many such cases will have returned to rehabilitation in a separate episode. Multiple episodes for the same individual were not linked in this analysis, but the process of concatenation will support linkage of serial episodes in future analyses.

Based on these criteria, the dataset was divided into 28,596 complete and 8076 incomplete episodes as shown in the Fig 1. Table 1 shows the categorisation of these included case episodes by AROC Impairment code. The profile was broadly similar although the incomplete group included a slightly higher proportion of traumatic brain and spinal cord injuries.

### Grouping of condition according to NSF categories

**Fig 2:**
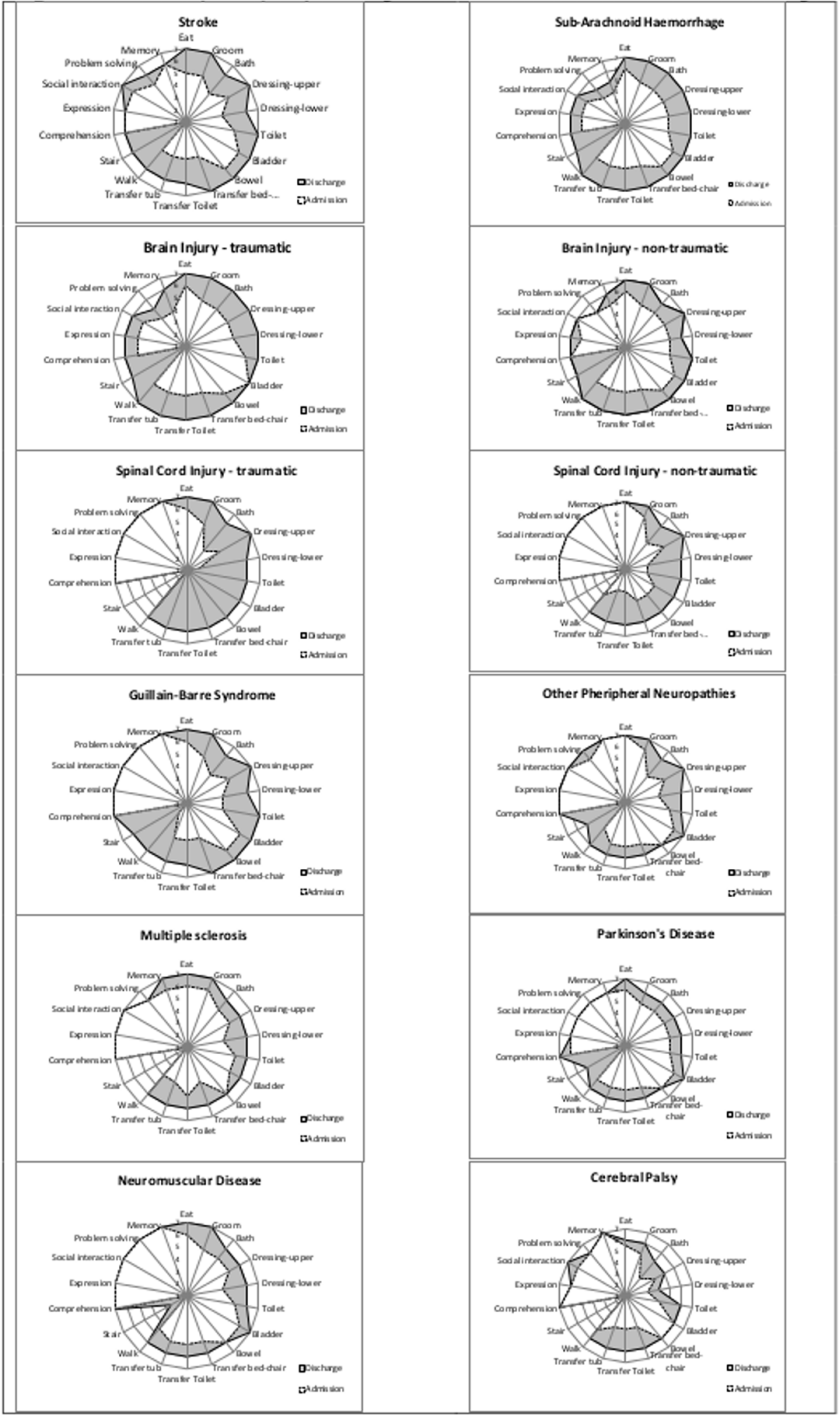
Radar Charts (FIM-Splats) showing median FIM scores on admission and discharge for the 12 groups. The FIM-Splat provides graphic presentation of the disability profile in a radar chart. The 18 items are arranged as ‘spokes of the wheel’ and the Levels from 1 (total dependence) to 7 (total independence) run from the centre outwards. Thus a perfect score would be demonstrated as a large circle. The group median scores for each item are plotted for admission and discharge. The difference between median scores on admission and discharge is depicted by the shaded area.

We first explored the extent to which episode data for the different neurological conditions could be collated into groups according to the NSF definitions. In an initial stage exploration, we separated the dataset for complete episodes into 12 condition categories as shown in Table 2. CP was the only identifiable condition within the ‘Stable’ category (there is no AROC impairment code for Post-polio). The 12 categories were then compared to determine whether similarities between them would support further collation under the three main NSF groupings. In addition to analysis of between-group differences in FIM total and subscale score, we examined visually the graphic presentations of the disability profile (FIM-Splats) for each of the 12 categories[13]. The FIM-Splat is a radar chart showing the median scores on admission and discharge for each of the 18 FIM items. Three assessors independently examined the 12 FIM-Splats, grouped them on the basis of observed similarities, and then conferred to reach consensus. Six main groups were identified for the final analysis (see Fig 2). These were collated into two broad NSF categories (‘Sudden onset’ and ‘Progressive /stable’ conditions) due to the small size of the Stable group.

**Table 2:** Grouping for analysis.

	12 groups		6 groups
NSF Category	Group	N =	Group
Sudden-onset	Cerebrovascular Accident	12527	Stroke
	Subarachnoid haemorrhage	1014	Other acquired brain injury (ABI)
	Brain injury—traumatic	4351	Other acquired brain injury (ABI)
	Brain injury—non-traumatic	2200	Other acquired brain injury (ABI)
	Spinal cord injury—traumatic	1677	Spinal cord injury (SCI)
	Spinal cord injury—non-traumatic	2076	Spinal cord injury (SCI)
	Guillain-Barré Syndrome	805	Guillain-Barré Syndrome (GBS)
Progressive	Multiple sclerosis	2292	Progressive
	Parkinson’s disease	847	Progressive
	Neuromuscular disease	408	Progressive
	Other (chronic) polyneuropathies	203	Progressive
Stable	Cerebral Palsy	196	Cerebral Palsy (CP)
	**Total**	28596	

### Outcomes of interest

The primary outcomes of interest were change in patient functional status, hospital length of stay (LOS) and discharge destination. In a more detailed analysis, we explored the specific items of function within the FIM (self-care, bladder/ bowel continence, mobility, cognition etc.) that did and did not change within each of the 6 groups.

### Statistical Analysis

There is continued debate over the appropriate methods for statistical analysis, in view of concerns about excessive mathematical manipulation of ordinal data[14]. Rasch analysis offers the theoretical option of creating interval level data from ordinal scales, but as yet there is a lack of consensus in how to achieve this. Rasch models are not yet available for every condition, and neither are bedside computers to provide instantaneous interval-level conversion. In routine practice, clinicians must interpret the ordinal data as best they can. To make this analysis meaningful for them, we have taken a simple pragmatic approach to this analysis using standard descriptive and non-parametric methods.

Descriptive analysis included the counts, percentage, mean, standard deviation, median and inter-quartile range as appropriate for demographic, LOS and discharge destination (percent discharged to community/remaining in hospital system) collated by condition. As age and LOS are interval data, between group comparisons were determined by one-way unrelated ANOVA with post hoc Bonferroni correction.

Previous studies have reported mean FIM gain and FIM efficiency (FIM gain/ length of stay)[2, 3], so these figures are given for the sake of comparison. However, as the FIM is an ordinal scale, we preferred the more conservative approach of using non-parametric statistics for analysis of FIM data[15].

- Spearman Rank tests were used to explore the association between Admission FIM scores and LOS.
- Within group comparisons of functional status (FIM) on admission and discharge were tested by Wilcoxon signed rank tests, for individual items, as well as subscales and total scores.
- Overall between-group comparisons were determined by Kruskal-Wallis tests. Post hoc group-by-group comparisons were undertaken using Mann Whitney tests. P values were multiplied by the number of tests to correct for multiple comparisons. Corrected p values of <0.05 were considered significant.

### Ethics Statement

This study was approved by the Human Research and Ethics Committee, The Royal Melbourne Hospital (HREC 2010.004)

## Results

Because nearly 22% of the sample comprised ‘incomplete’ episodes, it was pertinent to examine the characteristics of these cases, which are presented in Table 3. Overall the incomplete episode cases were younger (mean difference 2.2 years p<0.0001) than the completed group, with a greater proportion of males (64.9 vs. 60.8, Chi squared 41.7 p<0.0001) and had a longer length of stay (mean difference 3.8 days, p<0.0001). Over half (57.8%) were transferred back to acute hospital settings, 4.5% self-discharged at their own risk and <1% died during their IPR admission. Unsurprisingly, the majority of incomplete episodes were in the sudden onset conditions, particularly brain and spinal cord injury, where a higher incidence of serious inter-current illness is expected. The relatively high proportion of patients returning to the acute sector in this sample may reflect the relatively early transition to rehabilitation following injury in Australia and also the lack of investigation facilities in many free-standing specialist rehabilitation units necessitating a formal discharge each time a patient is referred to another hospital for investigation or treatment.

**Table 3:** Characteristics of the incomplete episodes (n = 8076).

	Stroke	ABI	SCI	GBS	Progressive	CP	Total
No. of Episodes(%)	2934 (36.3)	2618 (32.4)	1669 (20.7)	142 (1.8)	661(8.2)	52(0.6)	8076 (100.0)
Male N (%)	1845 (62.9)	1718 (68.0)	1223 (73.3)	83 (58.5)	287(43.4)	23 (44.2)	5242 (64.9)
Age—Mean (SD)	53.6 (10.2)	41.2 (14.6)	43.1 (14.9)	48.7 (13.7)	50.5(10.6)	35.0(12.8)	46.9 (14.1)
Length of stay, days—Mean (SD)	32.6 (42.1)	45.7 (72.1)	54.1 (64.3)	35.6 (61.7)	21.4(31.2)	16.4 (18.9)	40.3 (58.6)
**Discharge destination N(%)**							
Home/community	562 (19.2)	511(19.5)	150 (9.0)	40 (28.2)	202(30.6)	27 (51.9)	1492 (18.5)
Acute Hospital	1800 (61.3)	1411 (53.9)	1035 (62.0)	75 (52.8)	323(48.9)	20 (38.5)	4664 (57.8)
Own risk	168(5.7)	123(4.7)	25 (1.5)	8(5.6)	37(5.6)	0(0.0)	361 (4.5)
Care / other	198(6.7)	288(11.0)	240 (14.4)	8(5.6)	38(5.7)	4(7.7)	776 (9.6)
Died	31(1.1)	18 (0.7)	8(0.5)	1(0.7)	10(1.5)	0(0.0)	68 (0.8)
Missing	175(6.0)	267 (10.2)	211 912.6)	10 (7.0)	51(7.7)	1(1.9)	715 (8.9)

The remainder of the analysis includes only case episodes for a completed programme of rehabilitation (n = 28,296), divided into the six main groups. Demographics are shown in Table 4. The large majority of stroke, brain injury and GBS patients (>90%) were admitted from acute hospital services, with less than 10% coming from home. By contrast over 40% of patients with Progressive conditions and CP were admitted from home. In the spinal cord injury group, over three quarters came from hospital, but the rest were admitted from home or other non-hospital settings. Similarly, the large majority of patients were discharged back to their usual accommodation at the end of their rehabilitation programme (90.1% overall), although a significant proportion of sudden onset conditions (8.4% overall) were discharged to interim and other accommodation, compared with 4% in the progressive and stable conditions.

**Table 4:** Demographics within the six groups for the complete episodes (n = 28,596).

	Stroke	ABI	SCI	GBS	Progressive	CP
**Number of Episodes**	12527	7565	3753	805	3750	196
**Admitted from:**	Missing data n = 439 (1.5%)					
Home/community	1097(8.9)	526(7.0)	541(14.7)	44(5.5)	1453(40.0)	98(50.0)
Hospital	11077 (90.0)	6882(91.3)	2887 (78.4)	738(92.6)	2142(59.0)	97(49.5)
Other (eg care)	137(1.1)	131(1.7)	255(6.9)	15(1.9)	36(1.0)	1(0.5)
**Discharge to:**	No missing data					
Usual accommodation	11401 (91.0)	6607(87.3)	3277(87.3)	751(93.3)	3541(94.4)	188(95.9)
Interim accommodation	824(6.6)	822(10.9)	365(9.7)	48(6.0)	155(4.1)	4(2.0)
Other	302(2.4)	136(1.8)	111(3.0)	6(0.7)	54(1.4)	4(2.0)
**Gender** (%)	Missing data n = 32 (0.1%)					
Male	61.9	67.5	69.0	55.9	38.2	43.4
Female	38.1	32.5	31.0	44.1	61.8	56.6
**Age**	No missing data					
Mean (St Dev)	54.4(9.6)	42.6(14.9)	44.0(14.5)	47.1(13.4)	51.4(10.6)	40.6(13.8)
Median (IQR)	57(50–62)	45(29–56)	46(32–57)	50(38–59)	54(45–60)	41(29–52)
Range	16–65	16–65	16–65	16–65	17–65	16–65
**Length of stay (LOS), days**	No missing data					
Mean (St Dev)	34.6(31.4)	36.2(45.2)	56.9(59.5)	35.1(36.2)	22.9(21.5)	21.1(12.8)
Median (IQR)	25(14–45)	22(13–41)	36(17–75)	22(14–42)	17(12–26)	18(12–26)
Range	5–480	5–499	5–494	5–313	5–419	5–93
Spearman correlation LOS vs Total FIM score on admission	-0.655(p<0.001)	-0.627(p<0.001)	-0.559(p<0.001)	-0.667(p<0.001)	-0.410(p<0.001)	-0.255(p<0.001)
**FIM gain**	No missing data					
FIM-Motor gain (Mean + 95%CI)	21.6(20.3–21.9)	19.9(19.5–20.4)	20.2(19.6–20.8)	26.9(25.7–28.3)	11.8(11.4–12.2)	9.5(8.2–10.9)
FIM-Cognitive gain (Mean + 95%CI)	3.5(3.4–3.6)	5.5 (5.4–5.7)	0.6(0.5–0.7)	0.9(0.8–1.1)	0.9(0.8–1.1)	0.9(0.6–1.3)
FIM-Total gain (Mean + 95%CI)	25.1(24.8–25.5)	25.5(24.9–25.9)	20.8(20.2–21.4)	27.9(26.6–29.3)	12.8(12.4–13.2)	10.5(9.0–11.9)
FIM Efficiency (FIM total gain/LOS)	0.73	0.70	0.36	0.79	0.56	0.50

IQR = inter-quartile range; CI = Confidence interval; FIM = Functional Independence Measure.

Gender and age distribution were largely as expected—the predominance of females in the Progressive group being largely due to the preponderance of MS patients. All groups spanned at least the age range 17–65 years. One-way ANOVA tests confirmed significant between group differences in age (p<0.001). Post hoc analysis with Bonferroi correction confirmed significant differences between all groups except ABI and CP (p = 1.000).

Mean length of stay ranged from 21 to 57 days. One-way ANOVA tests confirmed significant between group differences in length of stay (p<0.001). Post hoc analysis with Bonferroi correction confirmed three distinct groups:

- SCI (Longer stay) (typically mean 8 weeks)
- Stroke, ABI and GBS (Medium stay) (typically mean 5 weeks)
- Progressive and CP (Shorter stay) (typically mean 3 weeks)

As expected there was a strong negative correlation between LOS and FIM total score on admission, but correlations were stronger for the four ‘Sudden-onset’ conditions (rho -0.56 to -0.66) than for the ‘Progressive/Stable’ conditions (rho -0.26 to -0.41). Gains were smaller for the progressive and sudden onset groups, but were proportionate to their shorter lengths of stay.

### Within groups analysis

Table 5 shows the median (IQR) for FIM total and subscale scores on admission and discharge, together with the results of Wilcoxon tests. All six groups showed statistically significant change (p<0.001) between admission and discharge in both motor and cognitive subscales as well as total scores.

**Table 5:** Median (IQR) FIM total and subscale scores on admission and discharge, with Wilcoxon Signed Rank tests for change between admission and discharge.

	Stroke	ABI	SCI	GBS	Progressive	CP	Total
	12527	7565	3753	805	3750	196	28596
**Functional Independence Measure (FIM)**	**MedianIQR**	**MedianIQR**	**MedianIQR**	**MedianIQR**	**MedianIQR**	**MedianIQR**	**MedianIQR**
**Admission Scores**							
FIM Motor	5737–74	6645–80	4426–66	5634–71	6143–73	5234–68	597–74
FIM Cognitive	2821–33	2316–29	3533–35	3533–35	3228–35	3126–35	2921–34
FIM Total	8561–103	9065–106	7759–100	8968–105	9272–106	8262–101	8663–104
**Discharge Scores**							
FIM Motor	8372–89	8880–91	7651–83	8378–88	7660–83	6943–80	8271–89
FIM Cognitive	3126–35	2924–33	3534–35	3535–35	3329–35	3328–35	3227–35
FIM Total	113100–120	116105–121	11084–118	118112–123	10890–116	9873–114	11399–120
**Change Scores**							
FIM MotorSign z score*	198-33z = -93.6	155-29z = -70.4	155-33z = -49.3	2411-41z = -24.2	94-17z = -49.0	71-14z = -10.9	166-30z = -138.9
FIM CognitiveSign z score*	20-5z = -74.1	40-9z = -63.5	00-0z = -17.0	00-0z = -12.3	00-1z = -25.4	00-1z = -6.2	10-5z = -103.7
FIM TotalSign z score*	2210-37z = -94.6	208-37z = -72.7	165-34z = -49.4	2412-42z = -24.3	104-19z = -49.2	82-15z = -11.0	188-34z = -140.9

*All significant at p<0.001.

### Between groups analysis

**Fig 3:**
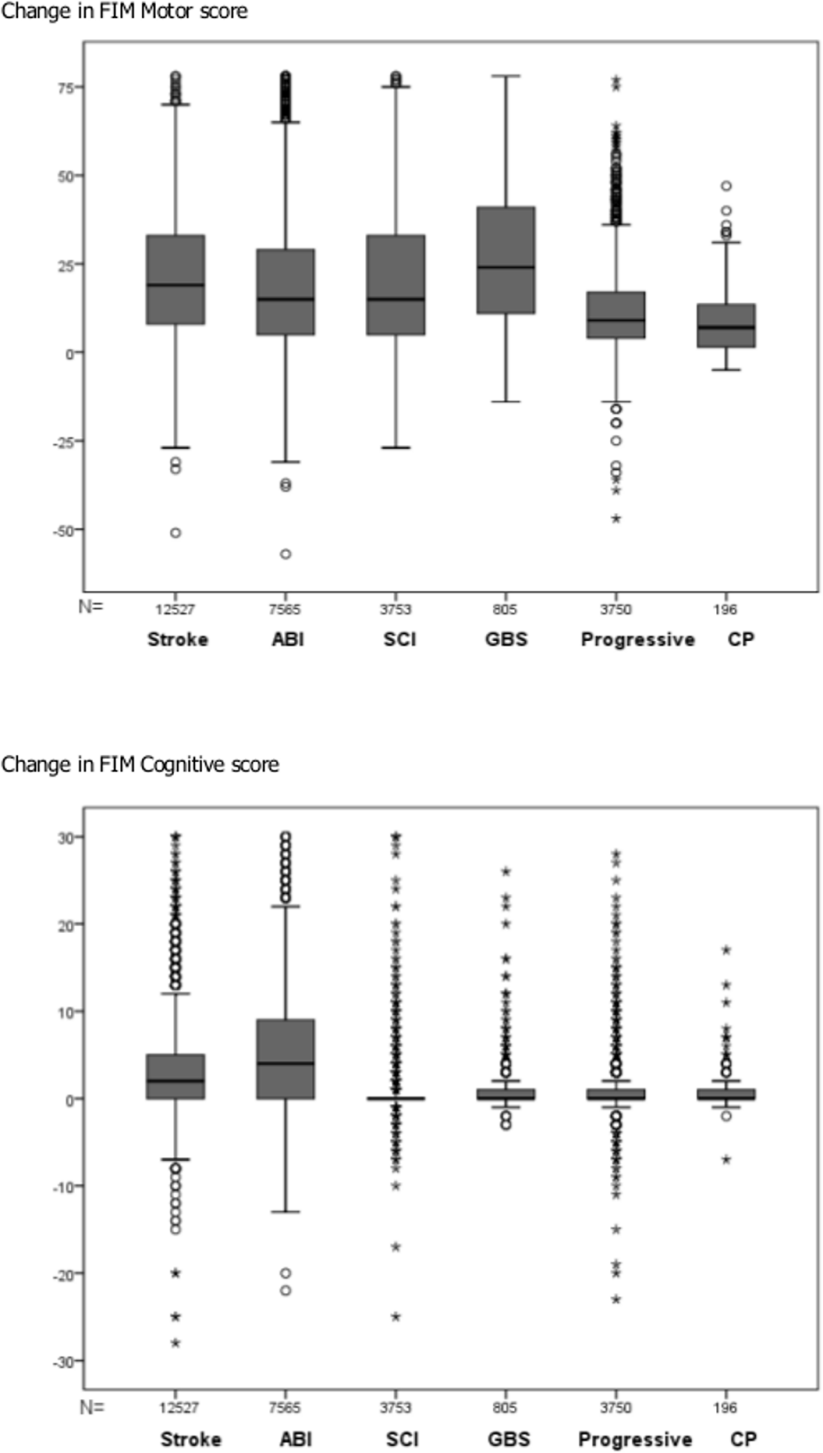
FIM Motor and Cognitive change scores. Medians are denoted by solid black lines while the top and bottom box edges denote the first and third quartile. The T-bars or whiskers denote the largest and smallest data within 1.5 times the interquartile range. The small circles are outliers, values that do not fall in the inner fences. The extreme values amongst outliers are marked with an asterisk. These are cases that have values more than three times the height of the boxes.

Fig 3 illustrates the FIM motor and cognitive change scores between admission and discharge for the six groups. Kruskal-Wallis tests confirmed significant overall between-group differences (p<0.001) for both FIM-Motor and FIM-Cognitive change scores. Post hoc Mann Whitney tests for change in Motor function confirmed significant differences between all groups (corrected p<0.01), except between ABI and SCI (p = 1.000). Post hoc tests for change in Cognitive function similarly confirmed significant differences between all groups except between GBS, progressive conditions and CP (p = 1.000).

### Item by item analysis

All FIM items showed statistically significant change in all conditions (Wilcoxon p<0.001). However the size of the change was often small. Table 6 shows the analysis of group level data and records the improvement (between admission and discharge) in median FIM score for each item in each of the six groups.

- In the FIM motor items, all groups showed improvement of 2 or more points across at least two items. The Progressive group showed smaller changes, but nevertheless change was seen across all motor items except sphincters, despite the relatively short lengths of stay.
- In the FIM cognitive items, the stroke and ABI groups showed improvements of 0–2 points, but there was no improvement for SCI or GBS.

**Table 6:** Improvement in the median FIM item scores between admission and discharge.

FIM Items	Stroke	ABI	SCI	GBS	Progressive	CP
**Motor**						
Eating	2	1	0	1	1	1
Grooming	2	2	1	2	1	1
Bathing	2	**2**	2	2	1	2
Dress-up	2	1	2	2	1	1
Dress-low	2	2	**4**	2	2	1
Toilet	2	2	**4**	**3**	1	2
Bladder	1	0	**4**	1	0	0
Bowel	1	1	**4**	1	0	0
Bed transfer	**3**	2	**3**	**3**	1	2
Toilet transfer	2	2	**5**	2	1	2
Tub transfer	2	2	**5**	2	1	2
Walk	2	2	1	**4**	1	1
Stairs	**5**	**5**	0	**5**	2	0
**Cognitive**						
Comp	0	1	0	0	0	0
Express	0	1	0	0	0	0
Social	1	1	0	0	0	1
Problem	1	1	0	0	0	0
Memory	0	2	0	0	1	0

Change in median value: in bold >2.

**Fig 4:**
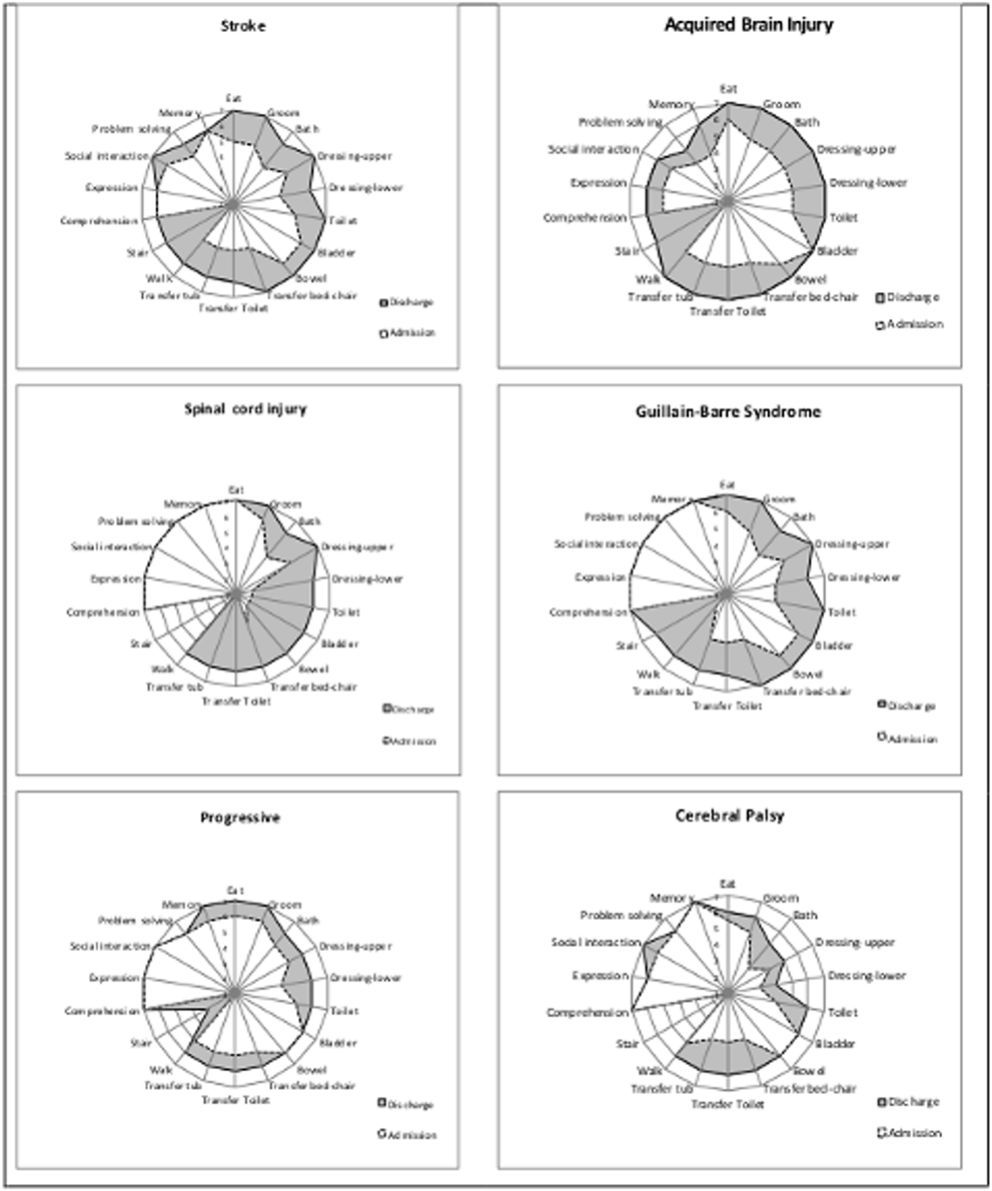
FIM—Splat Motor and Cognitive change score. The FIM-Splat provides graphic presentation of the disability profile in a radar chart. The 18 items are arranged as ‘spokes of the wheel’ and the Levels from 1 (total dependence) to 7 (total independence) run from the centre outwards. Thus a perfect score would be demonstrated as a large circle. The group median scores for each item are plotted for admission and discharge. The difference between median scores on admission and discharge is depicted by the shaded area.

Fig 4 shows the radar charts (‘FIM-Splats’) of the median item scores on admission and discharge for the six groups. The different pattern for each group is clearly seen. In SCI and GBS, the lack of change in cognitive scores reflects a ceiling effect at admission. Near-ceiling effects also account for the small degree of change in Progressive conditions. In CP, there were mild cognitive deficits at baseline, but these largely remained static.

## Discussion

In this analysis of a large Australian dataset, we have compared outcomes from in-patient rehabilitation across groups of long term neurological conditions, categorised according to the UK NSF for Long Term Neurological Conditions into Sudden-onset, Progressive and Stable Conditions. The analysis was centred on adults of working age to reflect the emphasis of the NSF, and also the predominant focus of many of the more specialised rehabilitation services in the UK[16].

The literature contains a number of other analyses from large multi-centre rehabilitation datasets–notably held by Uniform Data Systems in the USA which is undoubtedly the largest in the world. Previous reports (including other published analyses of the AROC dataset) have tended to focus on single conditions, such as stroke[2, 17, 18], traumatic brain injury[3], spinal cord injury[19, 20] multiple sclerosis[21, 22] or Guillain-Barré Syndrome[23, 24]. These have either provided a general description for benchmarking information[2, 3, 22, 23] or compare outcomes across different rehabilitation settings[17] or for different racial and ethnic groups[18]. Ottenbacher et al (2004) examined year-on-year trends in length of stay, living setting, functional outcome, and mortality[25]. Several authors have used Rasch analysis to examine differential item functioning (order of difficulty for individual items) across different neurological conditions[26, 27] and smaller single centre analyses from the UK have examined changes in FIM for a general neurorehabilitation sample[28, 29]. However, this is one of very few large clinical dataset analyses from outside the US and the first to compare functional outcomes at item level across different neurological conditions grouped according to the NSF categories.

The data were collected in the course of routine clinical practice and we have deliberately kept the analysis simple, so that clinicians can interpret it and use it to compare their own practice. FIM-splats have proven popular in clinical settings, both in the UK and in Australia, for providing an ‘at-a-glance’ impression of the areas in which change has occurred. The use of FIM-splats to inform clinical grouping based on the profile of change across individual FIM items is a novel approach, which we believe will provide a useful basis for future analyses of the dataset. For example, small groups of rarer conditions may be included within the group with the closest-matching FIM profile. Alternatively, where the condition is to be considered separately, examination of the FIM profile may be used to inform the selection of an appropriate comparator group. This approach also may have potential application for other large datasets around the world.

Although the AROC dataset does not directly provide categorisation into the three main NSF groups, it was possible to group the conditions by impairment code. The logic of separating groups into the NSF categories was to some extent borne out by the analysis. At the crudest level there were clear differences between the ‘Sudden-onset’ conditions and the ‘Progressive’ and ‘Stable’ conditions in terms of the source of admission, length of stay, discharge destination and functional gain. However to group the conditions into just two or three main categories would miss important differences between them. Between groups analysis, together with examination of the different patterns of improvement as shown in the FIM-Splats, suggests that the six-group analysis performed here represents an appropriate balance between capturing clinically important differences and maintaining a manageable number of groups for statistical comparison.

Within groups analysis showed that all groups made statistically significant changes in FIM score between admission and discharge, at the levels of total, subscale, and individual item scores.

- All six groups showed substantial changes in motor function. Even at item-level, all groups made gains that were likely to be clinically important (see below) across a wide range of items.
- With respect to cognitive and communicative function, Stroke and ABI patients showed moderate change, and the Progressive and CP groups showed smaller changes. However, the majority SCI and GBS patients were already at the upper limit of the scales on admission.

Across all domains and all conditions the scores may go either up or down between admission and discharge, as illustrated in Fig 2. Even where the neurological condition does not directly affect the brain (i.e. spinal cord injury, GBS), a proportion of patients do have problems in the cognitive domains (for example due to inter-current infection, metabolic disturbance or occult brain pathology), which have a general impact on function and are addressed during the rehabilitation process. Therefore, as other authors have highlighted[20, 30], the absence of change in FIM cognitive score may represent a ceiling effect of the scale itself, rather than a genuine lack of change. A number of solutions have been proposed, including the addition of further items to address cognitive / psychosocial function, to form the Functional Assessment Scale (FIM+FAM)[31, 32] Whilst the FIM+FAM may not significantly extend the scaling range of the FIM, there is evidence that it provides extended coverage of individual goals for rehabilitation on a qualitative level[33].

We recognise a number of specific limitations to this study.

1. As with analysis of any large dataset collected in the course of routine practice, there was significant attrition due to incomplete data. Although data are carefully checked and validated at the point of submission to AROC, and completeness of data entry is improving over time[22], the possibility of missing data, coding and reporting errors still exist, which could affect the results.
2. The AROC dataset records de-identified episodic data, resulting in the potential for more than one episode being reported against the one patient with all but one being incomplete. However, since 2013 calendar year AROC has introduced a new analysis practice called ‘Concatenation’. Prior to outcomes analysis, AROC will identify groups of submitted episodes that can be joined to form a single AROC reporting episode, and consequently this will reduce the overall proportion of incomplete cases in the dataset.
3. The AROC dataset was not designed to separate episodes according to the NSF categories, and we recognise that the division between groups is not entirely clean. Similarly, as noted elsewhere[22], the AROC dataset does not distinguish the different patterns of onset of MS. The dataset did not provide robust enough information about the overall duration of the condition to separate these longer-standing patients with confidence, so cases were allocated to groups of the basis of the AROC impairment code alone. There are further opportunities for future analysis, for example to compare outcomes for patients admitted from acute services and from the community.
4. In such a large dataset even small differences are likely to reach statistical significance, even if they are of no clinical importance. A key challenge for this type of analysis is therefore to define what is meant by ‘clinically important’ change.

In terms of crude change in FIM score, the findings in this study are on par with other reports. For example, Beninato et al 2006[34] reported changes of 17, 3 and 22 respectively in FIM motor, cognitive and total scores in association with a Minimal Clinically Important Difference (MCID) in stroke. Our study showed changes of this order for the ‘Sudden onset’ conditions (see Tables 4 and 5). To our knowledge, MCID for FIM has not been reported for progressive conditions.

More importantly, however, the theoretical implication of improved independence following rehabilitation is that there should be a corresponding reduction in care needs, and therefore on-going costs in the community. The quantification of cost-benefits is a key challenge for any prospective data collection system. Granger and colleagues in the USA have reported a change of 1 point on the total FIM scale to equate to approximately 5 minutes of care per day for TBI patients[35], 3.32 minutes for stroke[36] and 3.38 minutes for MS[21]. Although it cannot be assumed that care costs translate across different health and social care cultures, were we to apply these estimates, the mean change in FIM recorded in this series (see Table 4) would equate to a reduction of approximately 14.8 care hours per week for TBI, 6.3 hours/week for stroke and 5.0 hrs per week for MS. However, this analysis is over simplistic, as Granger himself also points out. Some FIM items are more predictive of care requirement than others, and this may vary across the different conditions. For example, in MS locomotion and tub transfers were the strongest predictors[21], whereas for TBI, cognition and the need for support to maintain safety was a key factor[35]. At the very least, therefore, item level analysis is required to understand the impact of rehabilitation within the different conditions. Further analysis is on-going with this dataset to examine interval level changes and differential item functioning using Rasch and other techniques, and will be reported separately.

### Implications for future data collection

Within the AROC dataset, the FIM serves a multifunctional role. The admission FIM score is applied as a casemix tool, the level of dependency being used as a proxy indication of need for rehabilitation and care. Change in FIM score from admission to discharge is reported as the primary outcome measure, and FIM efficiency (FIM gain / length of stay) is reported as a surrogate for service-efficiency. Although this model has the advantage of simplicity and minimising the burden of data collection, particularly in high throughput services, it may be too simple to provide adequate evaluation in the context of complex neurological rehabilitation. Data gathered in a tertiary neuro-rehabilitation setting in the UK demonstrate that, although the FIM correlates fairly well with needs for care and nursing, it is a poor predictor of needs for therapy and medical intervention[37]. Moreover, due to floor and ceiling effects, ‘FIM efficiency’ was shown not to be a sensitive indicator of cost-efficiency, other than in the middle part of the score range[38]. There are also concerns about the validity of this type of mathematical manipulation of ordinal data[14].

Other measures are therefore required to provide a more complete evaluation of the complexity of ‘needs’ for rehabilitation as well as the ‘inputs’ (in particular staff resources) provided to meet them, before we can properly interpret measures of outcome and cost-efficiency. Over the last decade or so, newer tools have been developed and validated in the UK to provide more direct evaluation of these aspects. The Rehabilitation Complexity Scale[37] is a simple measure of rehabilitation needs. The Northwick Park nursing and therapy Dependency Scales[39–41] are measures of dependency which translate directly into estimates of staff time via a computerised algorithm, and have been used to provide a more direct evaluation of cost efficiency, especially for more dependent patients[38]. These are now incorporated into the UKROC dataset, as well as the option of recording the UK FAM items[32] to provide more comprehensive evaluation of cognitive and psychosocial outcome for those centres who wish to record them. The AROC dataset is also under review and the next iteration could potentially include a somewhat extended dataset to capture some of these parameters. Both centres are also exploring methods for pseudonymising patients, in order to track them through the system. The establishment of a common core of information for inclusion in these and other national rehabilitation datasets around the world would assist future international collaboration in outcomes analysis for rehabilitation.

## References

[1] The National Service Framework for Long Term Conditions. London: Department of Health 2005.

[2] Granger CV, Markello SJ, Graham JE, Deutsch A, Ottenbacher KJ. The Uniform Data System for Medical Rehabilitation: Report of Patients with Stroke Discharged from Comprehensive Medical Programs in 2000–2007. Am J Phys Med Rehabil 2009;88:961–72. 10.1097/PHM.0b013e3181c1ec38 19935180

[3] Granger CV, Markello SJ, Graham JE, Deutsch A, Reistetter TA, Ottenbacher KJ. The Uniform Data System for Medical Rehabilitation: Report of Patients with Traumatic Brain Injury Discharged from Rehabilitation Programs in 2000–2007. Am J Phys Med Rehabil 2010;89:265–78. 10.1097/PHM.0b013e3181d3eb20 20299850 PMC2918420

[4] Horn SD, DeJong G, Ryser DK, Veazie PJ, Teraoka J. Another look at observational studies in rehabilitation research: going beyond the holy grail of the randomized controlled trial. Arch Phys Med Rehabil 2005;86:S8–S15. 16373136 10.1016/j.apmr.2005.08.116

[5] DeJong G, Horn SD, Conroy B, Nichols D, Healton EB. Opening the black box of post-stroke rehabilitation: stroke rehabilitation patients, processes, and outcomes.[see comment]. Arch Phys Med Rehabil 2005;86:S1–S7. 10.1016/j.apmr.2005.09.003 16373135

[6] Horn SD, Gassaway J. Practice-based evidence study design for comparative effectiveness research. Med Care 2007;45:S50–7. 17909384 10.1097/MLR.0b013e318070c07b

[7] Eagar K. The Australian National Sub-Acute and Non-Acute Patient casemix classification. Aust Health Rev 1999;22:180–96. 10662228 10.1071/ah990180

[8] Long Term Neurological Conditions Dataset. NHS Information Centre Leeds 2010.

[9] National Definition Set for Specialised Services. Third edition London: Department of Health 2009.

[10] Green J, Gordon R. The development of version 2 of the AN-SNAP casemix classification system. Aust Health Rev 2007;31 (suppl 1):s68–s78. 17402908 10.1071/ah070s68

[11] Heinemann AW, Linacre JM, Wright BD, Hamilton BB, Granger C. Relationships between impairment and physical disability as measured by the Functional Independence Measure. Arch Phys Med Rehabil 1993;74:566–73. 8503745 10.1016/0003-9993(93)90153-2

[12] International classification of functioning, disability and health Geneva: World Health Organisation 2002. Report No.: ISBN 91 4 154542 9.

[13] Turner-Stokes L. Outcome measures for in-patient neurorehabilitation settings—a commentary. Neuropsychol Rehabil 1999; 9:329–43.

[14] Merbitz C, Morris J, Grip JC. Ordinal scales and foundations of misinference. Arch Phys Med Rehabil 1989;70:308–12. 2535599

[15] Svensson E. Guidelines to statistical evaluation of data from rating scales and questionnaires. J Rehabil Med 2001;33:47–8. 11480471 10.1080/165019701300006542

[16] Core service specification for complex specialised in-patient neuro-rehabilitation services in London London: London Specialised Commissioning Group 2005.

[17] Deutsch A, Granger CV, Heinemann AW, Fiedler RC, DeJong G, Kane RL. Poststroke Rehabilitation: Outcomes and Reimbursement of Inpatient Rehabilitation Facilities and Subacute Rehabilitation Programs. Stroke 2006;37:1477–82. 16627797 10.1161/01.STR.0000221172.99375.5a

[18] Ottenbacher KJ, Campbell J, Kuo Y-F, Deutsch A, Ostir GV, Granger CV. Racial and Ethnic Differences in Postacute Rehabilitation Outcomes After Stroke in the United States. Stroke 2008;39:1514–9. 10.1161/STROKEAHA.107.501254 18340094 PMC2642622

[19] New PW, Simmonds F, Stevermuer T. A population-based study comparing traumatic spinal cord injury and non-traumatic spinal cord injury using a national rehabilitation database. Spinal Cord 2011;49:397–403. 10.1038/sc.2010.77 20603631

[20] Hall KM, Cohen ME, Wright J, Call M, Werner P. Characteristics of the Functional Independence Measure in traumatic spinal cord injury. Arch Phys Med Rehabil 1999;80:1471–6. 10569443 10.1016/s0003-9993(99)90260-5

[21] Granger CV, Cotter AC, Hamilton BB, Fiedler RC. Functional assessment scales: a study of persons with multiple sclerosis. Arch Phys Med Rehabil 1990;71:870–5. 2222154

[22] Khan F, Turner-Stokes L, Stevermuer T, Simmonds F. Multiple sclerosis rehabilitation outcomes: analysis of a national casemix data set from Australia. Multiple Sclerosis 2009;15:869–75. 10.1177/1352458509105230 19465445

[23] Khan F, Amatya B, Stevermuer T. Rehabilitation for Guillain Barre syndrome: Analysis of the Australian rehabilitation outcomes dataset. Journal Clinical Medicine and Research 2010;2:91–7.

[24] Alexandrescu R, Sieget RJ, Turner-Stokes L. Functional outcomes and efficiency of rehabilitation in a national cohort of patients with Guillain-Barré syndrome and other inflammatory polyneuropathies. PLoS One 2014;17;9:e110532. 10.1371/journal.pone.0110532 PMC4234218 25402491

[25] Ottenbacher KJ, Smith P, Illig SB, Linn RT, Ostir GV, Granger CV. Trends in Length of Stay, Living Setting, Functional Outcome, and Mortality Following Medical Rehabilitation. JAMA 2004;292:1687–95. 15479933 10.1001/jama.292.14.1687

[26] Dallmeijer AJ, Dekker J, Roorda LD, Schepers VP, Lindeman E, van den Berg LH, et al Differential item functioning of the Functional Independence Measure in higher performing neurological patients. J Rehabil Med 2005;37:346–52. 16287665 10.1080/16501970510038284

[27] Granger CV, Hamilton BB, Linacre JM, Heinemann AW, Wright BD. Performance profiles of the functional independence measure. Am J Phys Med Rehabil 1993;72:84–9. 8476548 10.1097/00002060-199304000-00005

[28] Freeman JA, Hobart JC, Playford ED, Undy B, Thompson AJ. Evaluating neurorehabilitation: lessons from routine data collection. J Neurol Neurosurg Psychiatr 2005;76:723–8. 15834035 10.1136/jnnp.2004.035956 PMC1739616

[29] Cano SJ, O'Connor RJ, Thompson AJ, Hobart JC. Exploring disability rating scale responsiveness II: do more response options help? Neurology 2006;67:2056–9. 17159121 10.1212/01.wnl.0000247664.97643.e8

[30] Davidoff GN, Roth EJ, Haughton JS, Ardner MS. Cognitive dysfunction in spinal cord injury patients: sensitivity of the Functional Independence Measure subscales vs neuropsychologic assessment. Arch Phys Med Rehabil 1990;71:326–9. 2327886

[31] Hall KM, Hamilton BB, Gordon WA, Zasler ND. Characteristics and comparisons of functional assessment indices: Disability rating scale, functional independence measure, and functional assessment measure. J Head Traum Rehabil 1993;8:60–74.

[32] Turner-Stokes L, Nyein K, Turner-Stokes T, Gatehouse C. The UK FIM+FAM: development and evaluation. Clin Rehabil 1999;13:277–87. 10460115 10.1191/026921599676896799

[33] Turner-Stokes L, Williams H, J. J. Goal Attainment Scaling: does it provide added value as a person-centred measure for evaluation outcome in neurorehabilitation following acquired brain injury? J Rehabil Med 2009;41:528–35. 10.2340/16501977-0383 19543663

[34] Beninato M, Gill‐Body KM, Salles S, Stark PC, Black-Schaffer RM, Stein J. Determination of the Minimal Clinically Important Difference in the FIM Instrument in Patients With Stroke. Arch Phys Med Rehabil 2006;87:32–9. 16401435 10.1016/j.apmr.2005.08.130

[35] Granger CV, Divan N, Fiedler RC. Functional assessment scales: A study of persons after traumatic brain injury. Am J Phys Med Rehabil 1995;74:107–13. 7710723

[36] Granger CV, Cotter AC, Hamilton BB, Fiedler RC. Functional assessment scales: A study of persons after stroke. Arch Phys Med Rehabil 1993;74:133–8. 8431095

[37] Turner-Stokes L, Williams H, Siegert RJ. The Rehabilitation Complexity Scale version 2: a clinimetric evaluation in patients with severe complex neurodisability. J Neurol Neurosurg Psychiatr 2010;81:146–53. 10.1136/jnnp.2009.173716 19587391

[38] Turner-Stokes L, Paul S, Williams H. Efficiency of specialist rehabilitation in reducing dependency and costs of continuing care for adults with complex acquired brain injuries.[see comment]. J Neurol Neurosurg Psychiatr 2006;77:634–9. 16614023 10.1136/jnnp.2005.073411 PMC2117444

[39] Turner-Stokes L, Tonge P, Nyein K, Hunter M, Nielson S, Robinson I The Northwick Park Dependency Score (NPDS): a measure of nursing dependency in rehabilitation. Clin Rehabil 1998;12:304–18. 9744666 10.1191/026921598669173600

[40] Turner-Stokes L, Shaw A, Law L, Rose H. Development and initial validation of the Northwick Park Therapy Dependency Assessment. Clin Rehabil 2009;23:922–37. 10.1177/0269215509337447 19779007 PMC2841519

[41] Turner-Stokes L, Nyein K, Halliwell D. The Northwick Park Care Needs Assessment (NPCNA): a directly costable outcome measure in rehabilitation.[comment]. Clin Rehabil 1999;13:253–67. 10392653 10.1191/026921599677787870

